# *In Vivo* Antiprotozoal Activity of the Chloroform Extract from *Carica papaya* Seeds against Amastigote Stage of *Trypanosoma cruzi* during Indeterminate and Chronic Phase of Infection

**DOI:** 10.1155/2014/458263

**Published:** 2014-09-08

**Authors:** Matilde Jimenez-Coello, Karla Y. Acosta-Viana, Antonio Ortega-Pacheco, Salud Perez-Gutierrez, Eugenia Guzman-Marin

**Affiliations:** ^1^Laboratorio de Biología Celular, CIR “Dr. Hideyo Noguchi”, CA Biomedicina de Enfermedades Infecciosas y Parasitarias, Universidad Autonoma de Yucatán, Avenida Itzaes No. 490 x 59, Centro, 97000 Mérida, YUC, Mexico; ^2^Departamento de Salud Animal y Medicina Preventiva, CA Salud Animal, Facultad de Medicina Veterinaria y Zootecnia, Universidad Autónoma de Yucatán, Carretera Mérida-Xmatkuil, Km 15.5 Carr. Merida-Xmatkuil, A.P. 4-116, Mérida, YUC, Mexico; ^3^Universidad Autonoma Metropolitana-Xochimilco, Calzada del Hueso No. 1100, A.P. 23-181, 04960 México, DF, Mexico

## Abstract

In order to evaluate the antiprotozoal activity of the chloroform extract of *Carica papaya* seeds during the subacute and chronic phase of infection of *Trypanosoma cruzi*, doses of 50 and 75 mg/kg were evaluated during the subacute phase, including a mixture of their main components (oleic, palmitic, and stearic acids). Subsequently, doses of 50 and 75 mg/kg in mice during the chronic phase of infection (100 dpi) were also evaluated. It was found that chloroform extract was able to reduce the amastigote nests numbers during the subacute phase in 55.5 and 69.7% (*P* > 0.05) as well as in 56.45% in animals treated with the mixture of fatty acids. Moreover, the experimental groups treated with 50 and 75 mg/kg during the chronic phase of the infection showed a significant reduction of 46.8 and 53.13% respectively (*P* < 0.05). It is recommended to carry out more studies to determine if higher doses of chloroformic extract or its administration in combination with other antichagasic drugs allows a better response over the intracellular stage of *T. cruzi* in infected animal models and determine if the chloroform extract of *C. papaya* could be considered as an alternative for treatment during the indeterminate and chronic phase of the infection.

## 1. Introduction

American trypanosomiasis (AT) also known as Chagas' disease is a neglected infectious disease caused by the hemoflagellate protozoa* Trypanosoma cruzi* (*T. cruzi*). The presence of the parasite is widely distributed in the American continent, from the south of the United States of North America to Argentina. AT is endemic throughout much of Mexico, Central America, and South America where an estimated 8 million people are infected [1].

Currently, the treatment protocols of Chagas' disease are based on the use of two drugs, nifurtimox, and benznidazole. Both drugs are considered inefficient, not only because of the narrow therapeutic range but also because of the associated toxicity. Natural products are considered an important source of biologically active compounds against various infectious organisms [2].

The* Carica papaya* (Linn) (*C. papaya*), commonly known as papaya, is a fruit crop cultivated in tropical and subtropical regions and well-known for its nutritional benefits and medicinal applications [3]. It is widely distributed in the south of Mexico including the Caribbean and grows at an altitude range of 10 to 1,600 m above sea level. The antimicrobial, antifungal, larvicidal [4], insecticidal [5], and antiprotozoal properties of* C. papaya* against* Trichomona vaginalis* [6] and* T. cruzi* [7] have been previously reported.

Particularly, the antiprotozoal activity of the crude chloroformic seed extract of* C. papaya* was observed from* in vitro* as* in vivo* studies during the acute phase of the infection [7]. The chemical composition of chloroform seed extracts of* C. papaya *has been previously reported by GC-MS, with oleic (45.97%), palmitic (24.1%), and stearic (8.52%) acids being the more abundant components [5]. The chloroform extract of* C. papaya* has no demonstrated toxicity when administered to different animal species (mice, rabbits, dogs, and monkeys) for periods of 30, 60, 120, or 300 days. It has been reported that the oral route administration of this extract does not induce significant changes in hematological values of treated animals showing any signs of toxicity caused by the administration of the extract [8–12].

In the present study the* in vivo* antiprotozoal activity of crude* C. papaya* seeds (extract and a mixture of main components) against* T. cruzi* intracellular replicative stage (amastigote) was evaluated during the subacute and chronic phases of the disease.

## 2. Materials and Methods

### 2.1. Plant Material and Crude Extract Elaboration

*Carica papaya* seeds from ripe fruits were collected from Escarcega, Campeche State, Mexico, through July to September of 2011. The plant was authenticated by Dr. Salvador Flores-Guido and a voucher (10284) was deposited at the herbarium of Universidad Autonoma de Yucatan (UADY). Seeds were dried at room temperature under shadow conditions. Fresh seeds from ripe fruits were shade-dried for 15 days and later coarsely powdered. In a 1 L bottom flask fitted with a reflux condenser, 100 g of dry ground papaya seed and 500 mL of chloroform were heated for 4 h, cooled to room temperature, and filtered. The solvent was dried under a vacuum in a rotary evaporator and then dried in a vacuum oven at room temperature for 12 h [5].

### 2.2. Parasites

Trypomastigotes of* T. cruzi* strain H4 were used for intraperitoneal (IP) inoculation. Parasites for inoculation were obtained from the blood of previously infected mice from where the strain is maintained. The selected strain (H4) was isolated in the Yucatan Peninsula area from a human clinical case and it has been described as a highly virulent strain [13] capable of producing a mortality rate of 50% in mice after 30 days of inoculation. Also the amastigotes of H4 strain have shown tropism to cardiac tissue and in a minor proportion of parasites invade skeletal muscle [14].

### 2.3. Animals

In order to evaluate the antiprotozoal activity of the chloroform extract and their main components during the subacute phase of AT, a total of 40 female BALB/c mice were infected with an IP inoculation of 5 × 10^2^ trypomastigotes (in 200 *μ*L of saline solution). Also, to evaluate the antiprotozoal activity of the extract during the chronic phase of the AT, another 32 female mice were inoculated with 1 × 10^2^ trypomastigotes. Mice included in the assays were 8 weeks old at day of inoculation.

The case definition for a subacute phase of Chagas disease was mice negative to* T. cruzi* at a parasitological test (Strout method or thick smear) inoculated at least 45 days before the evaluation and with the presence of positive IgG. The definition for chronic phase was mice with a negative* T. cruzi* parasitological test (Strout method or thick smear) inoculated at least 100 days before the evaluation and with the presence of positive IgG.

For the subacute phase evaluation, mice after 45-day postinfection (dpi) began to receive the extract during 15 days every 24 hours. The chloroform extract of* C. papaya* was administrated* per os *(PO) at the doses of 50 and 75 mg/Kg (*n* = 8 in each group). Before administration, the extract was mixed with phosphate buffered saline (PBS, 137 mM NaCl, 2.7 mM KCl, 4.3 mM Na_2_HPO_4_, and 1.4 mM KH_2_PO_4_, pH 7.4).

One group was treated with a mixture of the main fatty acids present in the extract (palmitic, oleic, and stearic acids) at a dose of 100 mg/Kg (*n* = 8). The proportion of each compound was estimated as a function of the percentage that each one of them is usually found in the crude chloroform extracts of* C. papaya* [5]. The main compounds from the chloroform extract of* C. papaya *tested in this research were obtained from a commercial presentation of every one (Sigma Aldrich codes: oleic acid O1008, palmitic acid P0500, and stearic acid 605581).

For the chronic phase bioassay, mice after 100 dpi began to receive the chloroform crude extract (50 and 75 mg/kg) during 15 days PO every 24 hours (*n* = 8 in each experimental condition).

For each bioassay, two control groups were considered. As a negative control (*n* = 8), a group of infected mice received only 50 *µ*L orally out of the vehicle (PBS), whilst a positive control group, including infected mice (*n* = 8), was treated orally with allopurinol (Sigma Aldrich, A8003) (8.5 mg/kg) diluted in 50 *µ*L of PBS every day during 15 days.

### 2.4. Evaluation of the* In Vivo* Antiprotozoal Activity against* T. cruzi* during the Subacute and Chronic Phase of the Disease

To determine the antiprotozoal activity against the intracellular amastigote form of* T. cruzi*, cardiac tissue samples from treated and untreated mice were collected and fixed in formaldehyde (10%). Afterwards, the paraffin-embedded tissue sections were stained with hematoxylin-eosin (HE) and examined under a light microscope. Four nonconsecutive slides from the heart of each mouse were also examined in a blinded mode.

The number of amastigote nests was quantified in 100 zones for each heart. All procedures were conducted in accordance with the internationally accepted principles for laboratory animal use and care [15].

In animals treated during subacute phase, the parasite burden was evaluated by absolute quantification with Quantitative Polymerase Chain Reaction (qPCR). Tissues (25 mg) were subjected to proteinase K lysis, and total DNA was isolated by chromatography columns, using the commercial kit DNeasy Blood and Tissue (QIAGEN, cat no. 69 506). Total DNA (100 ng) was used as the template in a real-time PCR (as described above) with oligonucleotides specific for a sequence encoding for satDNA of* T. cruzi* (TCZF 5′-GCTCTTGCCCACAMGGGTGC-3′; TCZR 5′-CCAAGCAGCGGATAGTTCAGG-3′) [16].

The amplification was conducted under the following cycling conditions after 15 min of denaturation at 95°C; PCR amplification was carried out for 50 cycles (95°C for 10 s, 55°C for 15 s, and 72°C for 10 s). Fluorescence data collection was performed at 72°C at the end of each cycle. After quantification, a melt curve was made with 74–85°C raising by 0.5°C each step and waiting for 4 seconds afterwards acquiring on green channel. Melting temperature (*T*_*m*_) of the amplicon was 81°C. Each 96-well reaction plate contained the standard curve and two negative controls. Negative controls consisted of a reaction with* T. cruzi*-specific primers without DNA or DNA extracted from the cardiac tissue of healthy mice. Each DNA sample was carried out in triplicate and* T. cruzi* load was estimated by the absolute quantification method.* CT* values for the* T. cruzi*-specific signal were normalized to GAPDH gene DNA levels.

### 2.5. Statistical Analyses

Data are expressed as means ± standard deviations (SD) and were derived from at least triplicate observations per sample (eight animals per group). Results were analyzed for significant differences by using analysis-of-variance procedures followed by Tukey's multiple comparison tests. The level of significance was accepted as *P* < 0.05.

## 3. Results and Discussion

The evaluation of the extract during the subacute phase with doses of 50 and 75 mg/kg and using 100 mg/kg of a mixture of the major fatty acid components (stearic, palmitic, and oleic acids) showed a good antiprotozoal activity against amastigote forms of* T. cruzi.*

**Figure 1:**
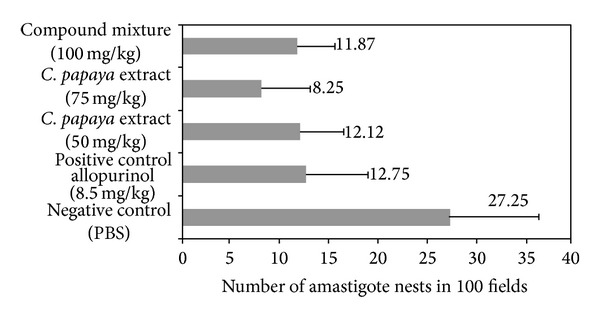
Effect of chloroform extract of seeds from* C. papaya *on the number of amastigote nests observed in cardiac tissue from mice BALB/c infected with trypomastigotes of* T. cruzi* and treated with 45 dpi at doses 50 and 75 mg/kg of chloroform crude extract and 100 mg/kg of the mixture of the fatty acids previously identified in the crude extract (*n* = 8 in each evaluated group). PBS and allopurinol (8.5 mg/Kg) were used as negative and positive control, respectively (*n* = 8 in each control group).

A reduced number of amastigote nests were observed in the cardiac tissue of infected mice during the subacute phase of the disease with the doses of extract evaluated (50 and 75 mg/kg), showing 55.5 and 69.7%, respectively (*P* > 0.05), compared with no treated group (negative control) (Figure 1). The mixture of the major fatty acid components exhibited an antiprotozoal activity of 56.45% similar to the crude extract evaluated doses and the mice group treated with allopurinol (positive control).

**Figure 2:**
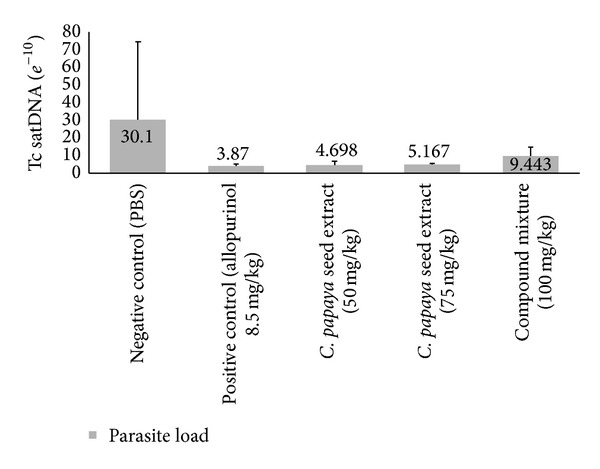
Effect of chloroform extract of seeds from* C. papaya* on the parasite burden (measured by qPCR) in DNA cardiac tissue from mice BALB/c infected with trypomastigotes of* T. cruzi *and treated with 45 dpi at doses 50 and 75 mg/kg of chloroform crude extract and 100 mg/kg of the mixture of the fatty acids identified in the crude extract. PBS and allopurinol (8.5 mg/Kg) were used as negative and positive control, respectively (*n* = 8 in each control group).

In agreement with the results recorded with histological techniques, when the parasite burden was measured by qPCR, a similar antiprotozoal effect in treated animals was observed, where a reduction of the parasite burden was 84.42, 82.86, and 68.64% (in comparison with the negative control group) for doses 50 and 75 mg/kg of crude chloroform extract and 100 mg/kg of the compound mixture, respectively (*P* > 0.05). However a total elimination of the DNA of the parasite was not observed in any of the doses tested (Figure 2).

**Figure 3:**
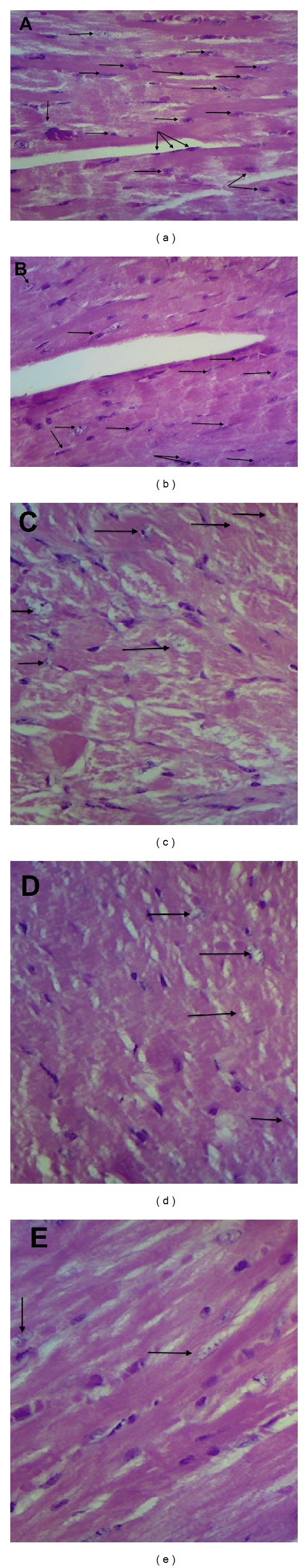
Microphotographs of cardiac tissue from BALB/c (40x, HE) during subacute phase of Chagas' disease. Untreated animals (negative control) (a), treated with allopurinol (positive control, 8.5 mg/kg) (b), treated with seed CE of* C. papaya* (50 mg/kg) (c), treated with seed CE of* C. papaya* (75 mg/kg) (d), and treated with the combination of FA (100 mg/kg) (CE = chloroform extract, FA = fatty acids, and arrows indicate the presence of amastigote nests).

During the examination of histological sections from the heart, a significant degeneration with severe diffuse coagulated necrosis in the cardiomyocyte cells was observed as well in the negative as in positive control group (Figures 3(a) and 3(b)). In the tissue of mice treated with 50 mg /kg dose of crude extract of* C. papaya*, a degeneration with severe diffuse coagulated necrosis, with a lower histiocytic infiltrate, was recorded (Figure 3(c)). Mice treated with a 75 mg/kg showed similar lesions and multifocal fibrosis (Figure 3(d)). Mice treated with the mixture of fatty acids showed necrosis with severe diffuse coagulated necrosis, histiocytic, lymphocytic, and moderated plasmacytic multifocal infiltration (Figure 3(e)).

After the analysis of the results, the most promising treatment option was chosen for the evaluation of the antiprotozoal activity during the chronic phase. This assay could be more complicated due to the long period (more than 100 days) required to reach the chronic phase and for ethical recommendations, and just the more efficient chloroform crude extract doses were assayed.

**Figure 4:**
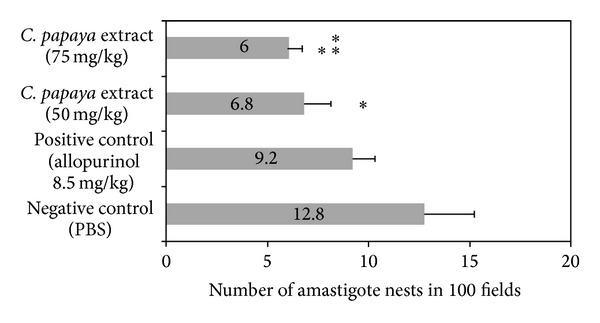
Effect of chloroform extract of seeds from* C. papaya* over the number of amastigote nests observed in cardiac tissue from mice BALB/c infected with trypomastigotes of* T. cruzi* and treated with 100 dpi at doses 50 and 75 mg/dg of chloroform crude extract (*n* = 8 in each evaluated group). PBS and allopurinol (8.5 mg/Kg) were used as negative and positive control, respectively (*n* = 8 in each control group) (*P < 0.05 versus negative control; **P < 0.05 versus positive control).

Results from monitoring of the doses of 50 and 75 mg/kg of the extract during the chronic phase confirmed the antiprotozoal activity of the evaluated extract. In mice treated with the extract (50 as 75 mg/kg) a significantly lower parasite amount (*P* < 0.05) was observed, projecting an 46.8 and 53.13% reduction in amastigote nests number compared to negative control group during chronic phase of the infection. Only the higher evaluated dose (75 mg/kg) showed statistically difference from positive control (*P* < 0.05) (Figure 4).

The use of* C. papaya* has been recurrent in the traditional medicine having a significant number of reports due to their properties for the treatment such as skin ulcers [17] and antiprotozoal activity against* Leishmania amazonensis* [18],* Trichomonas vaginalis* [6], and* Plasmodium falciparum* (*P. falciparum*) [19]. The antiprotozoal activity of the chloroform crude extract of* C. papaya *during the subacute and chronic phase of Chagas' disease is demonstrated in this study in concordance with previous reports [7].

Currently, the existing treatment for Chagas' disease is limited to only two drugs [2], being both toxic and with severe side effects. Natural products are an alternative source in the search for new drugs against* T. cruzi*. Also, the few treatment options tend to be subscribed during the acute phase of the disease; therefore, in the indeterminate and chronic stage of the diseases there is an urgent need for alternatives of treatment.

During the chronic phase of the disease, patients have different systemic pathologies predominantly at the cardiovascular and digestive level, and drugs used with adverse effects after consumption involve more risks than a potential benefit. Therefore it is important to search for alternative and effective options during the chronic phase (intracellular amastigote form) and no harmful to the infected individuals.

There are just few studies describing the antiprotozoal activity of natural products against amastigote form of* T. cruzi* and most of those investigations have been conducted under* in vitro* conditions [20–22]. In the performed bioassays, the* in vivo* antiprotozoal activity of chloroform extract of* C. papaya *seeds during the subacute and chronic phase of Chagas' disease was evaluated. During these stages of the disease, it is not feasible to determine the presence of parasites in the blood circulation (trypomastigote forms) and the only way to measure the antiprotozoal activity is to record the amount of the replicative intracellular form of amastigote, particularly in the cardiac tissue, because the H4 strain had previously shown high tropism there.

Some natural products have been described as active compounds against the amastigote stage of* T. cruzi* such as the ethyl acetate extract of* Piper jericoense*, in which one of its fractions (F4) showed significant activity against* T. cruzi* amastigotes yielding an IC50 of 56 *μ*g/mL and its selectivity index was 2.24 times higher than that of benznidazole. This fraction was reported as not cytotoxic, mutagenic, or genotoxic [20]. Another compound with antiprotozoal activity against the amastigote stage* in vitro *is the terpenoid hypnophilin, purified from* Lentinus strigosus*, which demonstrated a significant inhibitory activity over* T. cruzi *tripanotion reductase [22].

Finally another relevant report from natural products against* T. cruzi* was the one described by Veiga-Santos et al. [21], reporting piperovatine and piperlonguminine as compounds isolated from* Piper ovatum *which showed antiprotozoal activity against the* T. cruzi* amastigote stage. These compounds were able to cause severe alterations in the plasma membrane and cytoplasm of the parasite but show lower toxicity in mammalian cells. The* C. papaya *extract has not shown toxicity against mammal cells, but due to the antiprotozoal activity shown by the evaluated* C. papaya* seeds crude extract, it would be important to determine the possible targets in the parasite of this extract. The identification of differential proteins expressed between exposed and not exposed parasites to the evaluated extract allows for identifying the possible targets or metabolic routes involved in the antiprotozoal activity from the evaluated extract against* T. cruzi*.

The composition of the chloroform seed extract of* C. papaya* used in this study was previously determined by GC-MS and the oleic (45.97%), palmitic (24.1%), and stearic (8.52%) acids were the main components [4]. In other research conducted under* in vivo* conditions, but with an animal model infected with* Plasmodium *spp., the effect of the C18 fatty oleic, elaidic, and linoleic acids on malaria parasites was tested. These fatty acids inhibited the parasitaemia in mice infected with* Plasmodium vinckei petteri *or with* Plasmodium yoelii nigeriensis* in a 4-day suppressive test. After various experiments, this group concluded that fatty acids did not act at mitochondrial level of pyrimidine synthesis [23].

In another* in vivo* report, the antiprotozoal activity of* C. papaya* (crude aqueous extract obtained from leaves) against* Plasmodium berghei* has been reported. The combinations of artesunic acid and crude aqueous extract of* C. papaya* also prolonged the survival time of the infected mice and increased the recovery rate, compared to artesunic acid alone [24]. On the other hand, Pohl et al. [25] emphasized that fatty acids and their derivatives hold great potential as environmentally friendly antifungal agents or leads for novel antifungal drugs, and these facts could be partially shared with trypanosomatidae because some similar metabolic mechanisms are also present in these parasites.

For the case of* C. papaya* crude extract, it possesses a liquid consistency, and it reaches a concentration around 1 *μ*g/*μ*L; for that reason it is not feasible to evaluate higher doses in mice models. It is necessary to develop bioassays in other animal models (i.e., dogs) and to evaluate the antiprotozoal activity against* T. cruzi* at higher doses, as well as to evaluate the combination of different doses of the extract with specific antiprotozoal drugs (i.e., benznidazol) to determine whether any of these therapeutic options can result in a better response allowing the removal of the parasite.

The search for active compounds to identify new treatment alternatives for subacute and chronic phases of the disease is relevant, whether these new options may be useful to be used alone or in combination with other antiprotozoal drugs. Because AT is a neglected disease and pharmaceutical companies are not interested in searching for new drugs, there is a strong need to treat patients especially during the subacute and chronic phases of the disease.

Although administration of the chloroform extract of* C. papaya* seeds in the present study did not allow the complete elimination of the parasite during the subacute and chronic phases of the disease, these assays demonstrate a significantly positive effect in treated animals, particularly during the chronic phase of the disease, suggesting that better results could be obtained for antiprotozoal activity against the amastigote stadium when administered in combination with other antichagasic drugs.

The reduction of the parasite load during these phases of the disease may improve the prognosis and life expectancy of infected patients. For that reason, AT should be treated as a parasitic disease with the primary objective on reducing the parasite load in order to improve the effectiveness of the immune response and to reduce disease progression to cardiomyopathy [26].

## 4. Conclusions

The antiprotozoal bioactivity of extract obtained from the seeds of* C. papaya* has been shown to be effective against amastigotes of* T. cruzi* during subacute and chronic phase of infection in an animal model. However, it is necessary to evaluate higher doses if the assessed extract could have a better antiprotozoal activity when administered in combination with another drug which is currently used in the treatment of patients diagnosed with AT.
